# Perspectives on multimorbidity care provision among public hospital-based healthcare workers in Blantyre and Chiradzulu, Malawi: A qualitative study

**DOI:** 10.1371/journal.pone.0346493

**Published:** 2026-04-02

**Authors:** Gift Treighcy Banda-Mtaula, Ibrahim Simiyu, Sangwani Nkhana Salimu, Stephen A. Spencer, Nateiya M. Yongolo, Marlen Chawani, Hendry Sawe, Jamie Rylance, Ben Morton, Adamson S. Muula, Eve Worall, Felix Limbani, Miriam Taegtmeyer, Rhona Mijumbi, on behalf of the Multilink consortium

**Affiliations:** 1 Malawi Liverpool Wellcome Programme, Blantyre, Malawi; 2 Department of Clinical Sciences, Liverpool School of Tropical Medicine, Liverpool, United Kingdom; 3 Muhimbili University of Health and Allied Sciences, Dar es Salaam, Tanzania; 4 Kilimanjaro Christian Medical Center, Moshi, Tanzania; 5 KCMC University, Moshi, Tanzania; 6 Policy Unit, Malawi Liverpool Wellcome Programme, Blantyre, Malawi; 7 Kamuzu University of Health Sciences, Blantyre, Malawi; University of Kwazulu-Natal, SOUTH AFRICA

## Abstract

Multimorbidity, the presence of multiple chronic health conditions, is a leading cause of death globally. In Malawi, chronic noncommunicable and communicable diseases such as HIV frequently co-exist, putting pressure on an under-resourced system. However, the health system is primarily structured around disease-specific [vertical] programs, which hinders person-centred care approaches to multimorbidity. Our study focuses on multimorbidity care and explores the perceptions of healthcare workers on the patient pathways and service organisation throughout the patient’s interaction with the health facilities. This cross-sectional qualitative study took an interpretivist approach. We conducted 13 days of clinical observations at Queen Elizabeth Central Hospital and Chiradzulu District Hospital. We also conducted 13 days of clinical observations and semi-structured in-depth interviews with different cadres of purposively sampled healthcare workers (n = 22) at Queen Elizabeth Central Hospital and Chiradzulu District Hospital. Through thematic analysis, we identified an understanding of the organisation of care and healthcare workers’ perspectives on the delivery of services. Findings showed both hospitals provided services for inpatients and outpatients with multimorbidity, including screening, management, prevention of secondary conditions and rehabilitation. Patient diagnosis and management for multimorbidity were often delayed due to frequent stockouts of medication and consumables necessary for diagnostic testing for NCDs at the hospital level. Some healthcare workers were not equipped with the knowledge, skills, or guidelines to manage multimorbidity. As HIV care is currently better resourced than other chronic conditions, healthcare facilities may strengthen the supply chain, healthcare workers’ training sessions and monitoring and evaluation tools to ensure NCDs are well managed, learning from HIV programmes.

## Introduction

Multimorbidity, the presence of multiple chronic conditions, is a stressor to health systems, leading to poor quality of life, disability, treatment complications, and mortality [1]. In southern Africa, Roomaney, Kasambara and colleagues report a rising double burden of chronic communicable such as the human immunodeficiency virus (HIV) and non-communicable diseases (NCDs; e.g., hypertension). In Malawi, the health system is heavily dependent on donor funding for vertical (single-disease focused) health programmes, which leads to fragmented care for people with multimorbidity, that is inefficient and costly to the health system [2].

The World Health Organization (WHO) advocates for patient-centred care focusing on individual’s needs, values and preferences and integrated, holistic disease management for people living with multimorbidity (PLMM) [3]. Malawi’s Health Sector Strategic Plan III 2023–2030 reflects this commitment [4]. However, implementation is uneven and often relies on external funders. Integrated chronic care models, such as the Integrated Chronic Care Clinic (IC3) supported by Partners in Health, have demonstrated improved retention, clinical outcomes, and cost-effectiveness [5–7]. Yet, disease-specific guidelines and service organisation continue to constrain integrated multimorbidity management, contributing to poorly controlled chronic disease at hospital and community [8–11].

While existing qualitative studies have focused largely on patients’ and caregivers’ perspectives and experiences of living with multimorbidity [12–14], healthcare workers’ perspectives remain understudied despite their central role in shaping care delivery pathways and service organisation within facilities [15]. This study seeks to answer the question, “What are the perspectives of healthcare workers towards how clinical services are organised for patients with multimorbidity who present to the hospitals?” We defined multimorbidity as the presence of two or more of HIV, type 2 diabetes (T2DM), hypertension (HTN), and chronic kidney disease (CKD).

## Methods and material

This study was embedded within a multi-country prospective cohort study undertaken as part of the Multilink programme [16]. Multilink aimed to provide a comprehensive description of multimorbidity within secondary and tertiary care in Africa. Subsequently, it aimed to design and evaluate a complex intervention which identifies patients with multimorbidity during emergency assessment in hospitals in Malawi and Tanzania, optimising immediate treatment and ensuring post-discharge linkage to appropriate care [17].

### Study setting and population

We conducted this study in Malawi, a country in the southeastern part of Africa. Healthcare, at the point of service, is free at government-owned facilities. The study was conducted in two such public hospitals in the Southern part of Malawi: 1) Queen Elizabeth Central (QECH) hospital in Blantyre, which is the largest national referral hospital (1,350 bed capacity), 2) Chiradzulu District Hospital (Chiradzulu), a secondary facility (300 bed capacity) located 22.5 km from Blantyre which refers complex patients to QECH. Both sites are expected to provide multimorbidity screening, treatment, and follow-up.

We used purposive sampling to recruit healthcare workers directly involved in multimorbidity care in emergency, outpatient, and medical ward settings. Included cadres were consultants, medical officers, clinical officers, nurses, and pharmacists, selected for their complementary roles in diagnosis, treatment, continuity of care, and medication management. Participants were recruited to ensure variation in cadre, seniority, facility type, and experience. We only recruited licensed healthcare workers with at least 3 months of post-license work experience. Participant recruitment was based on convenience. Sample size was determined iteratively, guided by the principle of data saturation, defined as the point at which no new substantive themes emerged from additional interviews [18]. Saturation was assessed through ongoing analysis and comparison of interview data across cadres and facilities.

### Study design

This cross-sectional qualitative study adopted an interpretivist approach to explore how services for adults with multimorbidity are organised and experienced by healthcare workers [19]. We collected data through clinical observations and in-depth interviews with healthcare workers to triangulate the two data sources and gain a deeper understanding of participant experiences.

### Data collection

We collected data from 17 January to 23 August 2023. The lead author (GTB, a PhD candidate trained in qualitative research methods) conducted 13 days of semi-structured observations over 4 weeks, following an agreed structured observation guide [S1 Data]. The purpose was to iterate topic guides, understand the patient’s pathway and gain familiarity with the hospital context and get to know hospital staff. We made it clear to participants that these observations were conducted not to audit clinical service quality or assess appropriateness, but to understand structural and organisational processes that shape service organisation. We observed patient activities as they walked in and met a healthcare worker. We then selected patients [convenience sample] in the medical waiting area to observe their consultation sessions with healthcare workers to understand the interaction between healthcare workers. In the open spaces, patients were informed of our role by their treating nurse clinician. Outside of the open waiting areas, consultations with healthcare workers took place in private cubicles. Notes captured patient interactions, processes, how and when patients underwent registration, triage, and procedures for attending to patients that have been admitted; patient management, and availability of resources [20]. During clinical observations, we wrote down patient contact points. Clarifications were sought from the healthcare workers and patients being observed when required for further contextual understanding of the processes. GTB wrote observation summaries at the end of each observation session. GTB and FL identified and discussed the themes from the observation summaries. We developed an initial topic guide for in-depth interviews based on the components of the WHO health systems building blocks, concentrating on the service delivery building block [S1 Data] [21]. We then used the emerging themes from the observation summaries to expand the interview topic guide before piloting. The interview guide was piloted with three healthcare workers and was revised to improve clarity and flow. GTB (fluent in Chichewa and English) conducted semi-structured interviews face-to-face in a private room within the hospital facilities. The interviews were conducted in English, with participants combining some Chichewa phrases. Interviews lasted 30 minutes to an hour.

### Data management

Each participant was given a unique ID. Interviews were audio-recorded, transcribed, and translated into English where necessary. Transcripts were kept in a password-protected computer and a SharePoint folder accessible to Multilink study investigators.

### Data analysis

We conducted thematic analysis approach as described by Braun and Clarke [22]. The analysis was employed both deductively guided by the health systems dynamic framework (HSDF) with a focus on service delivery and human resources, and inductively by emerging data [23]. This was driven by our interest in the perspectives of healthcare workers and how systems shape MM care provision. The HSDF was used to examine how interactions between the WHO health system building blocks shape multimorbidity care provision.

Briefly, GTB, IS, and MT independently read transcripts to familiarise themselves with the data and coded the first three transcripts in parallel before developing a coding framework. Once the codebook was finalised, GTB coded transcripts in NVIVO 12 software [24]. Codes were reviewed and refined through constant comparison across transcripts. We constantly juxtaposed findings from observations and interviews to triangulate findings and gain a more comprehensive understanding of the organisation of care and its contextual aspects. We then charted the data to observe where patterns converged or diverged and grouped codes to identify emerging themes and sub-themes. We iteratively refined themes were iteratively and mapped them to the conceptual framework and study objectives.

### Quality assurance and reflexivity

Credibility and dependability were enhanced through triangulation, use of a consistent interview guide across sites, team-based coding discussions, and reflexive memoing. GTB and FL [fluent in English and Chichewa] independently matched English transcripts and audios to ensure quality and trustworthiness of the data. Additionally, participants were contacted by phone, and the context in which their quotes were used was read back to them, ensuring an accurate reflection of their perspectives in context [25]. We have reported this study in line with Consolidated Criteria for Reporting Qualitative Research (COREQ) checklist [S2 Data]. We have also provided an reflexivity statement on equitable partnership within the consortium [S3 Data].

The study team comprised clinical and non-clinical members, which increased the chances of understanding the data in multiple ways. The lead author completed her internship as a physiotherapist at Queen Elizabeth Central Hospital, which made her familiar with some of the clinical processes, workflow, and staff. Some clinician’s may have felt “inspected” by someone below or at a level with them in the medical hierarchy. Her role was not to determine whether a particular patient was appropriately managed but to review patient pathways. Even this was difficult at times, as these conditions are often complex and beyond her clinical area of expertise. Additionally, while some clinicians may not have the insight in recognising their limitations in managing such complexities, she may also have had the same lack of insight in areas that were less protocol-driven or pathways less clear. GTB used a semi-structured observation tool and topic guides and the team-based analysis to reduce bias.

### Ethical considerations

The study received ethical approval from the College of Medicine Research & Ethics Committee (COMREC) (Malawi) Ref P.11/21/3462 and the Liverpool School of Tropical Medicine Ref. 21–086 (UK). All interviewees gave informed consent. During observations, we obtained written informed consent from both healthcare workers and patients prior to observing consultation encounters. In situations where space was limited (such as bench waiting areas), we conducted observations only when privacy could be reasonably maintained. We did not collect patient-identifiable data. For the in-depth interviews, all participants signed a hard-copy consent form agreeing to be interviewed, to have their interview recorded, and to be anonymously quoted in dissemination activities.

## Results and discussion

We followed eight individual patient journeys from entry until they were discharged or admitted and allocated a bed. We conducted 22 interviews with healthcare workers with varied work experience, including the eight who were part of the structured observations (Table 1). One healthcare worker at Chiradzulu and three at QECH who were approached declined to participate in our study. These were replaced with consenting healthcare workers.

**Table 1:** Characteristics of study participants at Chiradzulu District Hospital and QECH(N = 22).

Category		Chiradzulu (n)	QECH(n)	Total (N)
**Gender**	Male	7	4	11
	Female	3	8	11
**Position**	Nurse	3	3	6
	Clinical Officer	5	2	7
	Medical Officer	1	4	5
	Medical Consultant	0	2	2
	Pharmacist	1	1	2
**Years of Experience**	0-4	2	6	8
	5-9	4	1	5
	10-14	1	3	4
	15-19	2	1	3
	20+	1	1	2

Findings illustrate how interactions between financing, service delivery models, workforce capacity and supply chain reliability for diagnostic equipment and medicines collectively shape the organisation of multimorbidity care. Findings are grouped into five major themes: Structural verticality and unequal integration, complex care needs for patient: Multimorbidity as a stress test of system capacity, provider capacity, and support to manage multimorbidity, resource scarcity and the reproduction of fragmentated care and system level opportunities to improve care.

### Structural verticality and unequal integration

**Fig 1:**
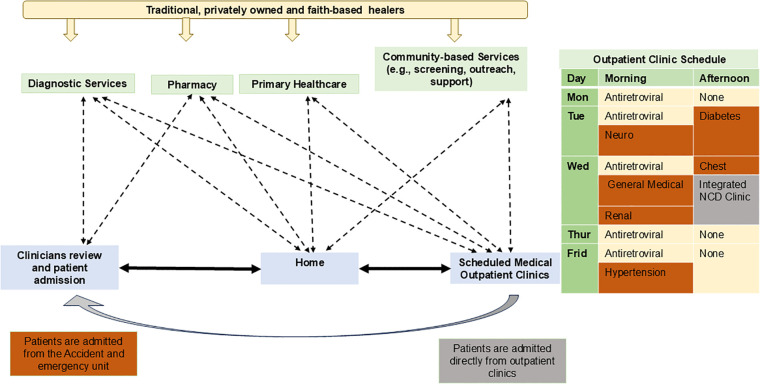
Organisation of care for PLMM linking to long-term outpatient clinics at the study facilities. Assigned colours: Deep yellow, Dusty Blue and Green: Services, Light yellow: Activities at both sites, Copper: Only at QECH, Grey: Only at Chiradzulu.

Healthcare workers described outpatient service organisation as both enabling and constraining for multimorbidity care. Although both sites provided multidisciplinary inpatient and outpatient care for chronic conditions, the structure and scheduling of clinics reflected historically disease-specific programmes, resulting in hybrid models of integration that varied by facility level and available resources. Fig 1 illustrates patient interaction between services and the scheduling of outpatient clinics for chronic conditions. At both facilities, patient pathways (also known as care maps) were similar to those of general medical patients until allocation to outpatient clinics where service organisation diverged.

### Routine outpatient clinics

At Chiradzulu District Hospital, clinicians described weekly integrated NCD clinic (HTN and T2DM only) alongside a daily Antiretroviral therapy (ART) clinic. Other NCDs apart from cancer (seen in palliative care clinics) were managed within general outpatient services. Clinicians reported patients with HIV were screened for NCDs within ART clinics but were referred to NCD clinics for management. At both site, Clinicians acknowledged an increase in the number of patients seen.

*“Initially we were booking about 50 patients but our clinics are still being overwhelmed so.”* [QECH_HCW05, female,10 years’ experience]

“…*currently, the numbers of patients with multimorbidity are going up and you can easily miss one or two things.*” [Chiradzulu, female,10 years’ experience]

At QECH, outpatient clinics for people not living with HIV were disease-specific, scheduled at different days and times [Fig 1]. Patients with multimorbidity were required either to attend multiple clinics or to rely on clinician discretion regarding which clinic would coordinate care. Clinicians perceived this structure as inefficient and burdensome for both patients and providers, limiting continuity and holistic care.

*“You’re booked a clinic for review and referred to all the departments that they think you could benefit from… But you are not guaranteed to get all services on the same day.”* [QECH, HCW06, male, 6 years’ experience]

The Lighthouse Trust (a centre of excellence in HIV management) at QECH offered daily integrated HIV + NCD clinics for stable PLHIV.

“*Lighthouse is a game changer for HIV, because they get everything done there…unless if the patient needs admission or specialist care, then they’ll send them (to specialist clinics).” [*QECH, HCW09, male, 4 years’ experience]

These scheduling arrangements interacted with staffing constraints and infrastructure limitations to produce repeated hospital visits and prolonged waiting times. For example, patients attending fasting blood glucose testing in the morning and waited until the afternoon for clinics, sometimes missed pharmacy hours. Fragmentation was not only a product of disease-specific care, but of temporal and logistical misalignment across departments. Clinic scheduling, pharmacy operating hours and staff availability operated as interconnected system components, shaping the patient experience of care.

*“You might be seen in the clinic, but find the pharmacy closed.” [QECH, HCW11, female, 4 years’ experience]*

Participants reported workforce shortages and high workloads constrained their ability to provide comprehensive assessments or adequately address patient needs. Workforce shortages led to reduced consultation time undermined integration because person-centred care not always feasible.

*“The (integrated NCD) clinic has more than 50 patients. They need to have vital signs and sugar levels checked, which takes time. When they go to a clinician, a full assessment takes time, and other people wait for a long time to be seen. So, we do not ask every patient their personal preferences.”* [Chiradzulu, HCW13, female,13 years’ experience]

### System redundancy in admission pathways

Differences in admission pathways further demonstrated how service organisation influenced patient burden. At Chiradzulu, patients requiring admission from outpatient clinics were sent directly to wards. At QECH, patients were referred to the Accident and Emergency and Trauma Centre (AETC) before admission. Clinicians described this as duplicative and frustrating for patients.

*“They are told to go to AETC for admissions, yet they are the same clinicians who will be admitting them.” [QECH, HCW01, female, 10 years’ experience]*

This requirement resulted in patients queuing twice, prolonging admission, and increasing workload. The redundancy reflects how emergency care protocols and outpatient systems function in parallel rather than as coordinated components, producing inefficiencies in multimorbidity management.

### Limited infrastructure and the experience of care

Physical space also shaped service organisation. QECH had dedicated outpatient clinic rooms located near the hospital entrance, which clinicians described as conducive to structured service delivery. In contrast, Chiradzulu’s integrated clinics shared space with general outpatient services, leading to crowding and reduced privacy.

“*There is a combination of patients attending the clinic and the general OPD, so there is disorganisation and lack of privacy. People don’t feel like they are at their own clinic*” [Chiradzulu, HCW16, Male, 5 years’ experience]

This infrastructure limitation interacted with patient volume and clinic design to affect confidentiality and perceived ownership of chronic care services. Integration at Chiradzulu occurred within spatial constraints that undermined the very continuity it aimed to promote.

### Value for integrated chronic disease management

There were conflicting views on the importance of integration for multimorbidity care. Most healthcare workers at Chiradzulu favoured integrated out-patient clinics, describing them as enhancing and broadening clinical competency.

*“Combined clinics are much better than having patients seeing different specialists because the condition can be managed at once, so monitoring them is easy and you [the clinician] also get better.” [*Chiradzulu, HCW13, female, 10 years’ experience]

In this setting, integration functioned as a workforce adaptation strategy. Given limited access to subspecialists, integrated clinics enabled generalist clinicians to consolidate care within a single encounter, reducing duplication and strengthening continuity. In contrast, clinicians at QECH, a tertiary referral hospital with greater specialist availability, often preferred specialised clinics. Here, integration was viewed as potentially diluting specialist expertise in complex cases.

*“…there are just certain nuances or certain small details for a patient who has complicated things that a specialist better manages.”* [QECH, HCW01, female, 10years’experience]

### Continuation of care and patient follow-up

Follow-up systems further reflected uneven institutional capacity. ART services routinely traced patients who missed appointments, supported by dedicated funding and staff. In contrast, NCD clinics lacked mechanisms for systematic follow-up, which left patient attendance unmonitored. This discrepancy illustrates how varying financing and governance structures for vertical diseases shape continuity of care. Consequently, continuity of care for PLMM was partially dependent on their HIV status.

*“I’ve never followed up patients with NCDs, I don’t even know who hasn’t showed up.* [QECH, HCW02, female, 3 years’ experience]

Clinicians described providing counselling and encouraged self-monitoring with personal BP machines and glucometers, drug adherence, dieting, physical activity, and general mental health and well-being. However, they acknowledged socioeconomic factors that limited the feasibility and were unattainable for some patients.

*“We guide them on doing physical exercises and self-tests. We encourage them to have their own glucometers and BP machines, but not everyone can buy the equipment.”* [Chiradzulu, HCW15, male, 4 years’ experience]

Community outreach occurred intermittently but was not systematically integrated into multimorbidity or chronic disease management. Referral to primary care facilities was common, yet clinicians expressed concerns about diagnostic capacity at that level, leading to delayed or missed diagnoses and acute presentations at hospital.

“*Sometimes a patient may present late to a hospital, not because she was not serious about her illness, but because she has been to various facilities [primary healthcare] and was misdiagnosed.” [QECH, HCW11, male, 25 years’ experience]*

### Multimorbidity as a stress test of system capacity

Healthcare workers consistently described multimorbidity not merely as the coexistence of diseases, but as a form of clinical and organisational complexity that amplified existing system constraints. PLMM required integrated decision-making, careful medication management, sustained lifestyle adjustments, and coordinated follow-up demands that frequently exceeded the design of disease-specific services.

Clinicians emphasised that common disease clusters, particularly HIV, HTN, T2DM and CKD, interact biologically, increasing the risk of further complications. This interaction increased diagnostic uncertainty and required anticipatory screening for complications which was further challenged by inconsistent supply of diagnostic tools.

*“T2DM, HTN and HIV predispose you to renal impairment and put you at increased risk of other comorbid conditions, including heart disease. As a clinician, you have to be aware of and screen for.” [QECH, HCW08, female, 4 years’ experience]*

However, the need for anticipatory and preventive management clashed with service models structured around curative single-disease clinics and time-limited consultations. In observations, none of the patients observed received health promotion or prevention talks.

### Polypharmacy and the limits of guideline-based care

Healthcare workers described polypharmacy as both unavoidable and burdensome. Managing multiple medications required careful consideration of drug-drug interactions and renal dosing. In observations, clinicians often asked patients about the drug history. Yet participants reported that national guidelines did not adequately address NCD-NCD multimorbidity.

*“Drug to drug interaction is challenging because you really need to think and the guidelines can’t cover everything.”* [Chiradzulu, HCW18, female,13 years’ experience]

While HIV guidelines were perceived as comprehensive in addressing ART interactions, equivalent guidance for NCD combinations was limited. In the absence of formalised protocols, clinicians relied on experience, peer consultation, or external online resources. Clinical complexity exposed a governance-service delivery gap that although integration was expected at the provider level, it was not sufficiently elaborated within guidelines. In this case, decision-making shifted from protocol-driven to individually negotiated which led to variability in care.

### Treatment burden and the negotiation of chronicity

Participants described the lifelong nature of chronic disease treatment as difficult for patients to accept. The requirement for sustained adherence across multiple medications compounded pill burden and psychological fatigue, in some cases led to a shift toward unregulated herbal medicines.

*“Patients need psychological support because if they have diabetes, hypertension, and HIV, they will sometimes ask if they should take all those drugs. Sometimes they lose hope and say it’s better to just die.” [*QECH, HCW03, female, 8 years’ experience]

Clinicians framed this not simply as patient non-adherence but as a misalignment between biomedical models of chronic disease and patient expectations of cure. In this context, unregulated herbal medicines were perceived as offering simplicity and finality, in contrast to indefinite pharmacological management.

*“People don’t understand that they will be taking the medication all their life… they prefer herbal medications that offer treatment once.”* [QECH, HCW02, female, 3 years’ experience]

Health education and counselling were routinely offered, but clinicians acknowledged that acceptance of lifelong treatment was uneven. The burden of chronicity in this case seemed to extend beyond clinical complexity into the social and psychological domains.

### Socioeconomic constraints and the feasibility of lifestyle modification

Multimorbidity management often required dietary modification, physical activity, and self-monitoring. However, healthcare workers recognised that recommended lifestyle changes were not equally attainable.

*“We advise them on what kind of food to eat and avoid; But not everyone can afford to change their meals.”* [Chiradzulu, HCW21, male,4years’experience]

Encouragement to purchase glucometers or blood pressure machines was perceived as aspirational for some patients.

*“We encourage them to have their own glucometers and BP machines. But not everyone can buy the equipment.”* [Chiradzulu, HCW15, male, 4 years’ experience]

These accounts demonstrate how structural poverty influenced clinical advice. The feasibility of “self-management” depended on household resources, meaning the burden of MM was unevenly distributed across socioeconomic lines. The complexity of MM was not only biomedical but was embedded within social and economic realities that shaped adherence and outcomes.

### Provider capacity and support to manage multimorbidity

#### Lack of senior support and limited communication.

Some clinicians felt unprepared to manage multimorbidity and reported lacking support in making clinical decision. Weak dissemination of existing policies and lack of clear MM guidelines led to reliance on informal WhatsApp peer consultations, which decentralised decision-making. Even at QECH, staff shortages meant subspecialists were *“not always available” t*o respond to queries, which challenged timely service delivery.

*“If a patient has HTN and CKD and we need the renal team to consult…it’s not easy for them to come maybe, because there’s only one [specialist].”* [QECH, HCW05, female, 10 years’ experience]

#### The need for on-the-job-training and refresher courses.

Uneven on-the job training sessions for HIV and NCDs left clinicians uncertain about managing the complexity (managing regimens, disease-disease interactions) of MM. Clinicians felt trainings could improve their capacity.

*“Just like they train people in managing HIV patients, if there is a new guideline, they train so we need the same in NCDs” [Chiradzulu, HCW16, male, 5 years’ experience]*

#### Resource scarcity and the reproduction of fragmented care.

Healthcare workers described institutional capacity constraints not as isolated operational gaps, but as structurally embedded conditions shaping how MM care is delivered. Workforce shortages, unreliable diagnostic systems, and medication stockouts interacted in ways that constrained clinical decision-making and reinforced vertical models of care. Multimorbidity complicated symptom interpretation, particularly where diagnostic capacity was limited. As a result, complexity at the patient level interacted with upstream resource constraints, contributing to late presentation and increased acuity at secondary and tertiary facilities.

#### Workforce constraints and adapting clinical practice.

Across both facilities, clinicians described chronic staff shortages, particularly limited availability of senior medical officers and specialists. Observations confirmed that much of frontline care was delivered by nursing students and junior clinicians. Participants explained that the scarcity of senior support did not merely delay consultations but shaped how clinical decisions were made and how care was prioritised.

*“…at the district hospital we have got few medical officers… At the same time, they are the ones with advanced knowledge, so it becomes a challenge when seeking second opinion.”*[Chiradzulu, HCW22, male, 25 years’ experience]

#### Managing complexity without institutionalised support*.*

In this context, MM management became heavily dependent on individual clinician experience rather than institutionalised protocols. Limited supervision reduced opportunities for shared decision-making in complex cases, pushing clinicians toward conservative or episodic management strategies. High patient volumes further compressed consultation time, limiting comprehensive assessment and reinforcing disease-specific encounters rather than holistic review.

“…*currently, the numbers of patients with multimorbidity are going up and you can easily miss one or two things.*” [Chiradzulu, HCW12, female,10 years’ experience]

Several participants described burnout and emotional strain, suggesting that workforce constraints affected not only technical capacity but also relational aspects of care. This dynamic undermined aspirations toward person-centred multimorbidity management, as clinicians prioritised immediate clinical stability over long-term coordination.

#### Diagnostic fragility and medical constrains.

Both facilities reported frequent breakdowns of diagnostic machines, stockout of testing and inconsistent availability of monitoring tools such as glucose sticks and batteries for blood pressure cuffs. During observations, at least one patient’s blood glucose couldn’t be measured because the glucometer was missing. Healthcare workers explained that such stockouts did not simply delay care but altered diagnostic pathways and constrained clinical reasoning. When laboratory or point-of-care testing was unavailable, clinicians either deferred decisions, relied on clinical judgement alone, or referred patients elsewhere, increasing patient burden and delaying management. In MM contexts where medication titration and monitoring are critical, diagnostic instability limited the feasibility of integrated management, leading to complications.

*“You may prescribe medication to find it’s not available at the pharmacy. Patients can go home without medication and end up with complications because they lack capacity to buy that medication.”* [Chiradzulu, HCW17, male, 14 years’ experience]

Frequent NCD stockouts structurally reinforced verticality in care delivery. As HIV commodities remained consistently available through donor-supported supply chains, clinicians were able to provide uninterrupted HIV care while NCD management became unreliable and availability-driven. This differential reliability implicitly prioritised HIV within routine practice, thereby reproducing disease silos despite policy commitments to integration. These challenges interacted with limited guidelines and biased funding structures to reproduce fragmentation, which was concerning, considering that both facilities acted as referral centres. This unequal availability of resources had systemic consequences, creating a cycle of acute presentations and hospital admissions.

*“We’re doing well in HIV because we have a lot of support from organisations, but we struggle with HTN and T2DM drugs…They come back with complications not because of suboptimal drugs, but because they’re not on one of their drugs.”* [QECH, HCW08, female, 4 years’ experience]

#### Loss of institutional trust.

The parallel supply chains reinforced disease hierarchies within routine practice which led to integration operating in structurally unequal systems. Even in facilities attempting integrated care, the dependable availability of HIV commodities and the instability of NCD medicines reproduced vertical service patterns and limited its effectiveness.

The lack of medication was felt to contribute to weakening institutional credibility and potentially drove patient disengagement from formal care.

*“Some patients complain that every time they go to the hospital, drugs are not available, and they say it’s just the same as staying at home because they are not helped. They would rather take herbal medicine”* [QECH, HCW05, female, 10 years’ experience]

#### Policy-practice gaps and decentralised decision-making.

Although national policies advocate integrated chronic disease management, participants reported unequal awareness of NCD-specific policies. While HIV protocols were widely known and readily available, guidance for NCD-NCD multimorbidity was perceived as limited or insufficiently detailed. Available guidelines did not cover complicated cases of multimorbidity, which pushed responsibility for integration from the health system to individual clinicians.

*“If they have CKD and T2DM and or HTN or liver cirrhosis, there is nothing that tells you what to do.” [*Chiradzulu, HCW20, female,14 years’ experience]

This shift of responsibility from system-level protocol to individual clinician judgement represents a decentralisation of integration. Multimorbidity management became experienced-dependent rather than system-supported. Such reliance on informal knowledge networks and international guidelines such as the British National Formulary [BNF] partially compensated for governance gaps but also introduced variability in care.

*“If the patient has CKD, and they are also on tenofovir (TDF) based ART regimen, we will need to switch them from the TDF to a renal friendly ART regimen. For HIV, the national guidelines have such, but for other NCDs we often use Google to see drug interactions.”* [Chiradzulu, HCW14, male, 5 years’ experience]

#### Poor multimorbidity reporting.

We observed data clerks were responsible for completing patient data at both sites. Even though clinicians reported listing all diagnoses in the patient’s health passport or file, hospital reporting systems captured diseases individually, with quarterly reports submitted by disease category. Multimorbidity as a construct, was not formally reported. This reinforced single-disease accountability structures, limiting visibility of multimorbidity and even NCDs within health information systems and reducing incentives for integrated service planning.

*“Feedback is given to the district management team and the clinicians who attend the NCD clinic at review meetings. These meetings don’t happen as frequently as the HIV meetings.”* [Chiradzulu, HCW16, Male, 5years’experience]

### System level opportunities to improve care

Where HIV services were supported by strong financing, stable supply chains, clear guidelines, and frequent review meetings, care was streamlined and reliable. In contrast, NCD services lacking equivalent structural reinforcement were characterised by uncertainty and intermittency. This asymmetry constrained efforts toward integrated multimorbidity management, despite policy intentions. Clinicians identified multiple leverage points within these interdependent system interactions, including standardised multimorbidity protocols, combined-pill regimens, and nurse-led integrated clinics.

#### Strengthening governance and clinical guidance for multimorbidity.

Participants consistently highlighted the absence of standardised multimorbidity protocols as a barrier to confident clinical decision-making. Developing and disseminating clear, context-appropriate multimorbidity guidelines, especially addressing NCD-NCD combinations and polypharmacy could reduce reliance on informal consultation and online resources.

“W*e need to harmonise our guidelines and practices so that clinicians based in the district should have the same access as clinicians at QECH.”* [QECH, HCW01, female, 10 years’ experience]

Participants emphasised the need for structured dissemination through continuous professional development (CPD), mentorship, and inclusion of non-clinical staff such as data clerks.

“*.... all nurses, clinicians and data clerks need to be trained.*” [Chiradzulu, HCW16, male, 5 years’ experience]

Embedding multimorbidity protocols within routine training and supervision mechanisms would institutionalise integration rather than leaving it to individual clinician discretion. This could strengthen governance-workforce interactions and support standardised management across healthcare facilities.

#### Expanding workforce capacity and collaborative practice.

Workforce shortages and limited specialist availability constrained integrated care. Healthcare workers recommended increasing the number of trained providers and expanding nurse-led integrated clinics beyond HIV, hypertension, and type 2 diabetes.

*“We can start nurse-led clinics for well-controlled patients because then it’s just follow-up and getting a refill of their chronic disease medication. Those not well-controlled can then go to clinicians’ clinics”* [*QECH, HCW06, male, 6 years’ experience]*

Nurse-led daily integrated clinics were viewed as a practical adaptation that could reduce scheduling fragmentation and improve accessibility for patients. However, participants emphasised that expansion of service models must be accompanied by training in multimorbidity management to ensure quality is maintained. These approaches would enhance interaction between human resources and service delivery components of the system.

#### Improving reliability of medicines and diagnostics.

Participants repeatedly underscored that integration efforts would be undermined without reliable diagnostic equipment and consistent availability of medication. Strengthening NCD supply chains and ensuring functional diagnostic infrastructure were viewed as foundational requirements for effective multimorbidity management. Several clinicians proposed the use of combination therapies where clinically appropriate, drawing from tuberculosis treatment models.

“…*it can* *help if we can have antihypertensives that combine 2 different active drugs in one tablet…It lessens the pill burden and fosters adherence*.” [QECH, HCW07, male, 6 years’ experience]

Such approaches could reduce treatment burden and improve adherence, while also simplifying prescribing in complex cases. However, participants recognised that medication innovation must be accompanied by stable procurement and distribution systems to have sustained impact.

#### Leveraging HIV infrastructure to support broader multimorbidity care.

Clinicians recognised the relative stability of HIV services exemplified by consistent medication supply, active patient tracing, and regular data review meetings, as a potential foundation for broader integration. Extending similar mechanisms to NCD services could improve continuity for patients with multimorbidity.

Participants did not advocate dismantling vertical HIV programmes but rather leveraging their infrastructure to reduce inequalities between disease streams. This suggests a pathway for incremental integration that builds on existing system strengths rather than requiring full structural redesign.

#### Strengthening community linkages and early detection.

Healthcare workers emphasised the importance of community sensitisation, outreach screening, and education on chronic disease self-management. Participants perceived that improved awareness could facilitate earlier detection and reduce late hospital presentations.

*“We can equip and collaborate with community workers and volunteers to screen for these conditions. They can liaise with health centres to manage these patients or refer them to higher facilities in good time.*” [Chiradzulu, HCW13, female,13 years’ experience]

Strengthening linkages between community services, primary care facilities, and hospitals was seen as essential to reducing reactive admissions and supporting continuity of care. However, clinicians also acknowledged that primary-level capacity must be reinforced to prevent inappropriate referral and delayed diagnosis and hospital-reliant management.

## Discussion

This study demonstrates that challenges in delivering care for PLMM in Malawi’s public hospitals are not merely operational gaps but reflect structural inequalities embedded within the health system. Although both facilities provide elements of integrated care, service organisation, financing streams, workforce development, supply chains, and guideline dissemination remain unevenly strengthened across diseases. Multimorbidity exposes these imbalances. As a result, integration is frequently enacted through clinician adaptation rather than institutional design, producing variability in care and reinforcing fragmentation despite policy commitments to person-centred approaches. These findings position multimorbidity not only as a clinical challenge but as a systems-level phenomenon shaped by dynamic interactions between governance, resources, and service delivery structures.

### Availability and accessibility of services

Both tertiary and secondary healthcare facilities provided care for patients with multimorbidity. However, the secondary facility lacked a strong integrated approach for individuals managing both HIV and NCDs. Our study participants perceived HIV-NCD screening and NCD-NCD integration at Chiradzulu, as an approach for optimal management of multimorbidity, highlighting the need for integrated screening protocols supported by governance and information systems [26,27]. In this setting, NCD-NCD integration only included T2DM and HTN, reflecting broader regional patterns where integration often falls under HIV, T2DM and HTN [28–31]. While prioritising these disease clusters is pragmatic due to their high prevalence, it subsequently risks reinforcing selective care pathways that overlook the growing complexity of multimorbidity, rendering them unresponsive to epidemiological needs.

The desire for integration varied by level of facility, reflecting differences in clinical capacity, professional roles, and institutional norms. Clinicians at secondary-level Chiradzulu preferred integrated clinics, aligning with findings from nurses across all levels of care in Australia, where integration was viewed to support skill development and continuity of care [32]. Similarly, participants in this study and clinicians in Ethiopia favoured integrated NCD-HIV clinics, perceiving integration as a mechanism for skills transfer and more holistic management [33]. In these settings, integration appears to function as a capacity-building strategy enabling clinicians to manage a wider range of conditions.

This is in contrast to our findings at the tertiary-level facility [QECH] where most clinicians believed specialised clinics are better for the patient because they offer more comprehensive care for each condition. The differences could stem from the tertiary facility having consultants enabling high-level clinical decision-making and continuity for complex cases. However, it contributes to fragmentation for patients with multimorbidity, as they must attend multiple outpatient clinics. This could be limited by the availability of specialist staff and high patient numbers. These contrasting perspectives suggest integration is not inherently superior but context-dependent, shaped by workforce structures and facilities expectations. Rather than serving as a universal solution, integration may be most appropriate in lower-level facilities where it enhances system flexibility and workforce capability, while tertiary settings may require coordinated but not fully integrated models that preserve specialists’ expertise for complex patients [1]. This could mean that integration may not be a “silver bullet” to manage chronic diseases. Producing care models that align with the dynamic interaction of facility-level expectations and workforce capabilities, could optimise care for PLMM across the health system.

### Workforce constraints and system adaptation

Clinicians felt ill-equipped and supervised to provide adequate integrated care for PLMM, exacerbated by high patient workload, insufficient training, and mentorship. These constraints undermine clinical confidence and continuity of care. Similar concerns have been reported across both high and LMIC, where healthcare workers consistently emphasise the need for targeted training, effective supervision, and strategies to recruit and retain skilled staff for continuity of care [32,34,35]. These constraints interact with clinical decision-making and resource availability, forcing providers to prioritise certain conditions and compromise holistic care and long-term coordination.

Our findings highlight persistent challenges in providing person-centred integrated multimorbidity care, reflecting broader system constraints rather than a lack of provider commitment. Other studies underscore the importance of person-centred care, especially among PLHIV and NCDs in the WHO African region [36]. Experiences from Eswatini and South Africa illustrate how patient-centred care can be operationalised through a decentralised drug distribution for PLNCDs and PLHIV, leveraging PEPFAR’s supported infrastructure [37]. According to Goldstein *et al*, these models are enabled by deliberate investments in staff training, data systems, supply chain, use of expert clients and supportive policies [37]. Until such efforts are implemented in Malawi, aspirations for person-centred multimorbidity can remain difficult to translate and sustain in clinical practice.

Leveraging existing HIV services to promote multimorbidity care without compromising the quality of NCD care has been feasible in this region [38]. Similarly, evidence indicates that integrating HIV and NCDs does not compromise HIV care or retention to care [31]. Importantly, integration can also create mutual reinforcement, whereby well-established HIV infrastructure may support NCD management, and NCD programmes may sustain HIV services in contexts of donor withdrawal. This highlights a potential pathway for sustainable, system-level integration.

Our study participants reported regularly offering counselling and health education for the patient and their carers, reflecting practices that empower individuals and engage families in chronic disease management. This can be part of the empowerment and engaging individuals and families with chronic conditions, which the WHO propose will improve outcomes through person-centred care [3]. This suggests that integration efforts are most effective when they simultaneously strengthen both clinical services and patients, providing educational knowledge and supportive environments.

### Resource scarcity as a driver of fragmentation

Similar to findings from Ethiopia, clinicians in our study perceived inadequate diagnostic capacity and frequent drug stockouts fundamentally constrained the provision of effective multimorbidity care [39]. Consistent with reports from PLMM in Malawi, recurrent stockouts were not only a practical barrier to treatment but also a source of frustration and disengagement from formal care and turn to unregulated herbal treatments [12]. While previous work emphasised healthcare workers concerns about losing personal credibility in the eyes of the patient due to frequent drug stockouts, our findings suggest a consequential loss of credibility in the health facility [40]. This represents a critical feedback loop in which supply chain failures weaken patient trust, reduce service utilisation, and potentially exacerbate disease progression [40,41]. Such loss of confidence may extend beyond PLMM, threatening the legitimacy of the health system more broadly and increasing the risk of delayed care, preventable complications, and adverse population-level outcomes.

### Availability and implementation of guidelines and policies

Mirroring findings in chronic respiratory diseases, awareness and use of national NCD policies was low, particularly outside HIV programmes, thereby undermining efforts at service integration [42]. Consistent with a 2022 systematic review by Kassa *et al*., this reflects a broader pattern across the African region in which NCD policies and guidelines exist but are poorly disseminated to healthcare providers [43]. As Maimela and colleagues argued, insufficient policy and guideline dissemination, combined with limited monitoring and evaluation, reinforces uneven clinical competence across disease areas [44]. Inadequate policy dissemination represents a failure of effective governance and service delivery, whereby policy intent isn’t implemented in clinical practice. This helps explain the self-reported knowledge gaps among healthcare workers, and the HIV-focused service delivery.

In contrast, evidence from Ethiopia suggests that the availability of clear, accessible national guidelines can actively enable HIV-NCD integration by legitimising integrated practice and reducing clinical uncertainty [33]. These findings indicate that policy development alone is insufficient; effective dissemination and implementation mechanisms are critical for translating integration agendas into routine care.

## Strengths and limitations

To our knowledge, this is the first study from Malawi reporting the perspectives of healthcare workers on services for adults living with multimorbidity. The complementary data collection methods and diversity of the interviewees enhanced the credibility of the data. However, our study focuses on healthcare workers at two government-funded hospitals and on a narrow range of common morbidities. We did not include primary healthcare facilities, which also provide care to patients living with multimorbidity. Our findings may not be generalised to facilities located in various regions of Malawi or at faith-based and for-profit healthcare facilities as points of referral. We excluded community-based healthcare workers, patients, and policymakers, limiting insights into community-level continuity of care and policy-level insights. Since some of the clinics reported delayed or misdiagnosis at the primary healthcare level, further studies may explore community linkages, explore and quantify service readiness and availability for multimorbidity care. Future research should incorporate these perspectives to get a better understanding of service organisation and utilisation.

## Conclusions

Multimorbidity in Malawi’s public hospitals reveal structural asymmetries within a health system historically organised around vertical, single-disease programmes. Although national policies promote integrated and person-centred care, uneven financing, workforce capacity, diagnostic reliability, medicine supply chains, and information systems constrain implementation. Integration is therefore often negotiated at the point of care, relying on clinician adaptation rather than institutional alignment.

Addressing multimorbidity requires coordinated system strengthening rather than isolated service reforms. Leveraging established HIV infrastructure offers a pragmatic pathway, but sustainable progress will depend on extending comparable institutional support to NCD services. Multimorbidity should thus be understood not simply as a clinical challenge, but as a systems-level phenomenon requiring aligned, system-wide responses.

## Supporting information

S1 Data Data Collection tools. (PDF)

S2 Data Consolidated Criteria for Reporting Qualitative Research. (DOCX)

S3 Data Consortium Reflexivity Statement. (DOCX)
