# Supplementary material for: Perspectives on multimorbidity care provision among public hospital-based healthcare workers in Blantyre and Chiradzulu, Malawi: A qualitative study
Source: PLoS One. 2026 Apr 2;21(4):e0346493. doi: 10.1371/journal.pone.0346493 (PMC13046104; doi:10.1371/journal.pone.0346493)
Supplement: S1 Data — (PDF) [file pone.0346493.s001.pdf]

S1 - Data collection tools

Guide for Observation of consultation with patients

Please complete this check list for consultations with patients. Approach a patient in the queue (or ask nurse to introduce you) and ask if you may accompany them. **(Some questions you will have to ask the patient directly (e.g. about triage happening before consultation)**

Guide for Observation of Hospital Processes

	**Patient 1**
Clinician/ nurse descriptor (red Supplemental file 1<br><br>shoes/braids etc)	
Brief description of patient<br><br>(gender, age)	
Whether triage is performed and who does that?	
Specific measurements performed<br><br>during triage	
Where is this information recorded?	
Whether the patient was told and<br><br>explained of the readings	
Whether the patient is given the opportunity to explain their problem to the clinician	

Guide for Observation of consultation with patients

Please complete this check list for consultations with patients. Approach a patient in the queue (or ask nurse to introduce you) and ask if you may accompany them. **(Some questions you will have to ask the patient directly (e.g. about triage happening before consultation)**

Whether test results are explained<br><br>to the patients	
What were the next steps to admission at the ward?	
How is the patient received at the ward (including allocation of a bed)?	
What further tests happen at the ward (this might require asking the patient in subsequent days)	
Any other information until the patient is discharged/ dies?	
**Describe engagement between the HCWs and patient, body language, eye contact, facial expression, and whether they were concerned.**	

Guide for interviews with HCWs responsible for patients with multimorbidity

Purpose

To gain insights on the healthcare workers' experiences in the management of patients with multimorbidity, from diagnosis, patient navigation and clinical management in the hospital.

Introduction to interview guide

My name is ……. I am a researcher working on the Multilink project under MLW/MUHAS/LSTM. The Multilink project seeks to test and design systems that identify patients with multiple chronic diseases when seeking emergency care in sub-Saharan African countries. The overall project aim is to improve early disease treatment, ensure better follow-up and improve the quality of life for people with multimorbidity.

Thank you for agreeing to participate in this interview and for taking the time to speak with us today. This interview will be recorded for reference; however, your name and any other identifying information will not be included in the data we record and reports. You may refuse to answer any question or choose to stop the interview at any time.

Demographics

	**Question**	**Response**
**1**	Sex	☐Male ☐Female
**2**	Education	☐Primary ☐ Diploma ☐ BSc/Degree ☐PhD

		☐Secondary ☐Certificate ☐ Postgraduate<br><br>☐Others Specify
**3**	Job role/position	

I would like to know a little bit about you and your work. Could you explain to me your position and responsibilities, and how long you have been in this position?

-Probe for how their day looks

Could you please tell me what multimorbidity means to you?

Can you please describe your involvement and responsibilities in the clinical management of patients with multimorbidity in this hospital?

-Probe about the day-to-day services/activities he/she provides

Clinical Management of Multimorbidity

I want to develop an understanding of the tools you use for clinical management of patients with multimorbidity in this hospital. This will help us understand how multimorbidity patient care can be strengthened to improve the quality of life. The multimorbid conditions of fucus (hypertension, diabetes mellitus, HIV, and chronic kidney disease)

Can you explain to me what tools/guides/protocols you use to help you manage patients with multimorbidity in the hospital?

-Probe to see if they are international, national, or local guidelines/tools

Do they cover single chronic diseases or multiple?

-Probe how these are used

Can you explain to me how decisions about which chronic disease conditions are treated first (ranking) among multimorbidity patients are reached?

-Probe for disease-disease or drug-drug interactions?

What difficulties do you face when making these decisions?

How useful are the available tools in helping you reach clinical decisions?

Please explain to me any challenges/gaps you see with the available tools for clinical management of patients with multimorbidity in the hospital. (How can these challenges be addressed to improve the clinical management of multimorbidity).

Based on your experiences, what suggestions do you have regarding the available tools for the management of patients with multimorbidity?

What equipment do you feel is needed to diagnose and manage patients with Multimorbidity?

What are the challenges with ensuring this equipment is available and functional

Are there clinical algorithms for health workers to use to manage patients with multimorbidity?

-If they find these algorithms useful

In your opinion and reflecting on your experiences, what would effective multimorbidity management look like?

Clinical Management Training

Can you please explain to me how you feel your medical training has prepared you to manage patients with multimorbidity.

What areas of medical training do you feel need improvement to address the challenges in multimorbidity patient care?

Apart from the formal medical training, what other relevant chronic disease management trainings have you attended to help you provide care to patients with multimorbidity?

-Probe for CPDs, short-course training, workshops

In your opinion, what areas of capacity building and training in the management of multimorbidity do you feel healthcare workers need to provide quality care to patients with multimorbidity?

What training/policy changes have happened at this facility to cater to patients with multimorbidity?

Are there any uncertainties or any areas where you feel unsure in the management of patients with multimorbidity

-Probe to explore gaps in expertise

Patient Navigation

Can you walk me through the different steps and processes patients with multimorbidity undergo from their admission to discharge in this hospital?

-For clinical staff to obtain information on how multimorbidity diagnosis is reached, treatment is provided, and patients are managed

Which of the above steps do you feel work well now, and why?

Are there any steps that you feel need improvement?

-Probe for why these steps need improvement

Can you tell me more about the services currently available for patients with chronic conditions

-Probe for chronic disease clinics, integrated screening, chronic disease awareness/education

In your opinion, what do you think patients with multimorbidity value the most or desire to see changed in the care they receive in the hospital?

HCW Feelings on Multimorbidity Patient/Caregiver

In your opinion, how do patients with multimorbidity feel about the current care provided in the hospital?

-Probe about costs, timeliness, acceptability, safety

Based on your experiences, what do you think patients with multimorbidity value the most or would wish to see happening as far as their care is concerned?

What do you think are the priorities of caregivers looking after multimorbid patients at the hospital? What do they like and dislike?

From your experience of managing patients with multimorbidity what needs (resources and support) specific to patients with multimorbidity do not apply to other patients?

How do you support patients with multimorbidity to self-manage their conditions at home?

-Probe for education sessions, referrals to PHC

Overall, how prepared do you think the health system is to meet these needs?

-Probe for areas of improvement

Conclusion – any comment? Or is there anything you would like to tell me?

Thank you so much for taking the time to speak with me today. I am grateful for your insights on how the clinical management of multimorbidity is reached in this hospital/facility. Your perspectives will be shared in aggregate with the Multilink team, who will then design interventions to support and improve the clinical management of patients with multimorbidity
