# Supplementary material for: Perspectives on multimorbidity care provision among public hospital-based healthcare workers in Blantyre and Chiradzulu, Malawi: A qualitative study
Source: PLoS One. 2026 Apr 2;21(4):e0346493. doi: 10.1371/journal.pone.0346493 (PMC13046104; doi:10.1371/journal.pone.0346493)
Supplement: S3 Data — (DOCX) [file pone.0346493.s003.docx]

# **S3 – Reflexivity Statement**

1. **How does this study address local research and policy priorities?**

Before starting the study, local priorities were determined at two levels:

Policy level - the Directorate of Chronic Diseases in the MOH was involved and supported application of this research funding. Subsequent presentations at the MOH technical working group identified priorities for the research.

Service delivery level - we engaged healthcare workers, groups of patients and their caregivers, and community leaders (through established community advisory boards in the local institutions) in discussing their priorities in management of multimorbidity hence informing the design of the study.

1. **How were local researchers involved in study design?**

The study comprises co-authors from Malawi (GTB, SNS, FL, ASM and MC), Tanzania (IS, NMY and HS) and England (BM, EW, JR and MT). We also have RM(Uganda) and SAS(England), who have ongoing experience residing and conducting research in Malawi. The senior authors both from high- and low-income countries, have substantial experience in conducting, leading and organising collaborative research among diverse research groups.

1. **How has funding been used to support the local research team?**

The acquired funding was used to support the local team with a salary or stipend, and covered all research costs. GTB,IS,SNS and NMY received this funding as part of their PhD studies at the Liverpool School of Tropical Medicine.

1. **How are research staff who conducted data collection acknowledged?**

The authorship contribution statement highlights the roles of all authors. GTB collected the data and is the lead author of this paper.

1. **Do all members of the research partnership have access to study data?**

Members of the Multilink Consortium had access to data hosted by the Malawi-Liverpool-Wellcome Programme data portal, in line with the Data Management Plan.

1. **How was data used to develop analytical skills within the partnership?**

MT, FL and RM, BM and EW continually supported GTB, aiming to develop a multidisciplinary approach to data analysis.

1. **How have research partners collaborated in interpreting study data?**

GTB analysed the preliminary data, and these analyses were reported back to the consortium at a collaborative workshop. Consensus on consolidated data was further developed through a series of meetings between GTB,IS, SNS, SAS, NMY, BM, EW and FL, and later GTB, FL, RM and MT.

1. **How were research partners supported to develop writing skills?**

Senior academics (FL, BM, RM, ASM, JR, MT, and EW) supported the PhD fellows (GTB, IS, SNS, SAS, NMY) in enhancing their writing skills. Fellows also received Academic Writing training provided by the NIHR.

1. **How will research products be shared to address local needs?**

The findings from this study have been presented to stakeholders including policymakers and patient representatives. The findings are also being published as open access. Further engagement with research leaders and healthcare workers is anticipated.

1. **How is the leadership, contribution and ownership of this work by LMIC researchers recognised within the authorship?**

Authors RM and FL worked as part of the senior authorship team in developing this manuscript, and their contribution has been recognised as joint senior authors. GTB led the development of the manuscript and is the first author.

1. **How have early career researchers across the partnership been included within the authorship team?**

We have included early career researchers based in Malawi and Tanzania (GTB, SS, & NMY) within the authorship team.

1. **How has gender balance been addressed within the authorship?**

We have seven female authors (GTB, SNS, NMY, EW, MT, MC and RM) and seven male authors (HS, IS, SAS, ASM, BM, JR, and FL).

1. **How has the project contributed to training of LMIC researchers?**

GTB, IS, SNS and NMY have been supported through their PhD studies. They have individually led research projects and manuscript writing.

1. **How has the project contributed to improvements in local infrastructure?**

The Consortium renovated parts of the study sites.

1. **What safeguarding procedures were used to protect local study participants and researchers?**

We followed the Malawi Liverpool Wellcome Trust safeguarding protocol for data collection and dissemination.
